# Association between descending pain modulatory system and cognitive impairment in fibromyalgia: A cross-sectional exploratory study

**DOI:** 10.3389/fnbeh.2022.917554

**Published:** 2022-09-29

**Authors:** Paul Vicuña Serrano, Maxciel Zortea, Rael Lopes Alves, Gerardo Beltran, Cibely Bavaresco Deliberali, Amanda Maule, Iraci L. S. Torres, Felipe Fregni, Wolnei Caumo

**Affiliations:** ^1^Post-graduate Program in Medical Sciences, School of Medicine, Universidade Federal do Rio Grande do Sul (UFRGS), Porto Alegre, Brazil; ^2^Laboratory of Pain and Neuromodulation, Hospital de Clínicas de Porto Alegre (HCPA), Porto Alegre, Brazil; ^3^Department of Psychology, UNISINOS, São Leopoldo/Porto Alegre, Brazil; ^4^Institute of Neurosciences, Universidad Catolica de Cuenca (UCACUE), Cuenca, Ecuador; ^5^Laboratório de Farmacologia da Dor e Neuromodulação: Investigacoes Pre-clinicas, Centro de Pesquisa Experimental (CPE), Hospital de Clínicas de Porto Alegre (HCPA), Porto Alegre, Brazil; ^6^Laboratory of Neuromodulation and Center for Clinical Research Learning, Department of Physics and Rehabilitation, Spaulding Rehabilitation Hospital, Boston, MA, United States; ^7^Pain and Palliative Care Service, Hospital de Clínicas de Porto Alegre (HCPA), Porto Alegre, Brazil; ^8^Department of Surgery, School of Medicine, Universidade Federal do Rio Grande do Sul (UFRGS), Porto Alegre, Brazil

**Keywords:** fibromyalgia, fibrofog, cognitive impairment, working memory, descendant pain modulation system

## Abstract

**Background:**

The successful regulation of sensory input to the central nervous system depends on the descending pain modulatory system (DPMS). For the effective regulation of sensory input to the central nervous system and behavioral responses to pain, the DPMS is required. Its connection to fibromyalgia (FM)-related cognitive dysfunction has not yet been investigated. Therefore, this study tested whether measures of verbal fluency, sustained attention, and short-term and working memory could distinguish FM patients from healthy controls (HC). Additionally, it investigated, using a standardized paradigm, the link between cognitive ability and the function of the DPMS in responders and non-responders to the conditioned pain modulation test (CPM-test).

**Materials and methods:**

We enrolled 21 HC women and 69 FM patients, all of whom ranged in age from 30 to 65. We employed scores from the Trail Making Test (TMTB-A) (sustained and divided attention), the Controlled Oral Word Association Test (COWAT) (orthographic and semantic fluency), and the Digits subtest of the Wechsler Adult Intelligence Scale (WAIS-III) as dependent variables.

**Results:**

A generalized linear model (GLM) adjusted by educational level revealed significantly lower scores in FM than HC on the Span digits forward, COWAT-orthographic, and TMTB-A. For FM patients, multilevel MANCOVA revealed that the cognitive performance of non-responders compared to responders to CPM-test showed lower adjusted scores in Span digits forward (Partial-η^2^ = 0.358, *P* = 0.001), Span digits backward (Partial-η^2^ = 0.358, *P* = 0.001), COWAT-orthographic (Partial-η^2^ = 0.551, *P* = 0.001), COWAR-semantic (Partial-η^2^ = 0.355, *P* = 0.001), and TMTB-A (Partial-η^2^ = 0.360, *P* = 0.001). The association between the cognitive tests and the DPMS is moderated by the serum level of brain-derived neurotrophic factor (BDNF). Additionally, these cognitive assessments had a positive correlation with antidepressant use and pain threshold. The cognitive assessments, on the other hand, were conversely associated with a life of quality.

**Conclusion:**

Based on these findings, it can be shown that HC performed substantially better on cognitive exams than FM did. They demonstrated a link between clinical complaints about attention and memory and decreased DPMS effectiveness. Additionally, they demonstrated that the BDNF is a moderating element in a potential relationship between the severity of cognitive impairment and DPMS dysfunction.

## Introduction

Fibromyalgia (FM) is a chronic primary pain condition characterized by generalized musculoskeletal pain, fatigue, non-repairing sleep, cognitive alterations, and depressive symptoms (Duruturk et al., 2015; Treede et al., 2015). Around the world, 2–5 percent of people are affected by it (De Souza and Perissinotti, 2018). Only 30% had job modifications, 23% had received disability compensation for incapacity, and over 50% had lost the ability to do their everyday duties (Duenas et al., 2016). In 50% of the FM population, significant cognitive deficits have been found (Katz et al., 2004). These issues comprise the “FibroFog” syndrome, which includes amnesia, mental activity blurring, sensory overload, and a decreased capacity for thought, information processing, or conversational following (Kravitz and Katz, 2015).

Despite the subjective burden of cognitive symptoms associated with FM, several studies assessing cognitive performance by standard neuropsychological tests demonstrated that their performance was comparable to that of healthy controls (HC). They observed these findings on tests of verbal memory (Leavitt and Katz, 2009; Kim et al., 2012), visual memory (Grace et al., 1999), short-term memory storage (Landro et al., 1997), attention (Landro et al., 1997), and visual memory (Grace et al., 1999; Miro et al., 2011). A previous study looked at how effort, pain, exhaustion, and sadness affected the cognitive impairment in FM. However, they had an impact on the ratings for attention and information processing speed (Bar-On Kalfon et al., 2016). On the other hand, according to further research, FM had cognitive impairment that was evident in their performance on measures of executive functioning, attention, processing speed, and memory (Santos et al., 2018; Wu et al., 2018). The mental tiredness brought on by exerting more effort to perform well on a particular test might be a variable contributing to the variety of results. This idea is at least somewhat supported by neuroimaging findings, which show that FM sufferers require more brain resources to complete the same task than healthy people (Bar-On Kalfon et al., 2016). Additionally, psychological comorbidities, including depression, anxiety, and insomnia, may exacerbate the negative effects of pain on cognition (Austin et al., 2001; Airaksinen et al., 2005; Castaneda et al., 2008). Additionally, they take analgesics, especially opioids, which may contribute to cognitive deficits (Ersek et al., 2004). Despite conflicting findings, the American College of Rheumatology (ACR), which included them in the current diagnostic criteria for FM, acknowledged the significance of cognitive function for FM (Wolfe et al., 2016). Nevertheless, there isn’t a standardized battery of neuropsychological tests for evaluating cognitive function in chronic pain. Hence, it has been suggested in the literature that the cognitive assessment of FM include tests to gauge executive functioning, complex psychomotor speed, attention, and working memory (WM) (Kravitz and Katz, 2015).

Although there is a growing corpus of knowledge about cognitive failure in FM, it is still not apparent what brain pathways underlie its pathogenesis. Brain areas implicated in affective responses to pain, including the rostral anterior cingulate cortex (ACC), medial prefrontal cortex (mPFC), and amygdala, have been discovered to have altered neural activation patterns (Yarns et al., 2022). These regions, which include the anterior cingulate gyrus, prefrontal cortex (PFC), nucleus accumbens, and hypothalamus, input the descending inhibitory networks and contribute to emotional and cognitive aspects of pain (Mercer Lindsay et al., 2021). Through ascending and descending projections, the periaqueductal gray (PAG) is a crucial component in the regulation and propagation of pain. Notably, abnormal cortical control may contribute to the maladaptation of the descending pain modulatory system (DPMS), and this mechanism may be relevant to chronic pain in general. This agrees with further studies that found altered functional connectivity between the mPFC and PAG in patients with musculoskeletal, neuropathic, and inflammatory chronic (Drake et al., 2021). The PAG propagates nociceptive and analgesic inputs in a bidirectional manner and can reduce or increase pain perception (Benarroch, 2008). Additionally, as descending pathways may prevent or promote the transfer of nociceptive information from the spinal cord, the PAG plays a significant role in both adaptive and maladaptive modulations of the pain experience (Tracey and Mantyh, 2007). The PFC, striatum, and hippocampus are related to the lateral and dorsolateral subregions of the PAG, which have been linked to executive function (Coulombe et al., 2016).

Studies in both clinical and pre-clinical settings have shown that cognitive dysfunction and pain intensity are associated (Van der Leeuw et al., 2016). However, a network of cortical regions, limbic system components, and the spine-bulbospinal loop control the mechanisms underlying the link between chronic pain and cognitive impairment. It is necessary to examine the relationship between psychophysical, neurochemical, and cognitive functions considering this complex interaction. The spinal-bulbar-spinal loop, which is triggered by ascending nociceptive inputs, is a component of the endogenous pain inhibitory system examined by the conditioned pain modulation test (CPM-test). In several musculoskeletal chronic pain diseases, such as myofascial pain syndrome, FM, and osteoarthritis, the intensity of descending pain inhibitory system (DPIS) dysfunction is connected to pain severity (Botelho et al., 2016; Caumo et al., 2016). Additionally, serum brain-derived neurotrophic factor (BDNF) levels have a positive correlation with the degree of DPMS dysfunction (Soldatelli et al., 2021). According to Ong et al. (2019), this neurotrophic factor is highly expressed in the PFC in chronic pain and is a crucial marker of neuroplasticity linked to structural changes in various cortical regions responsible for learning, memory, fear, and emotional responses (such as the hippocampus and amygdala) (Mazza et al., 2018).

This body of data shows a gap in the research about the relationship between the severity of symptoms affecting PFC neural networks and the effectiveness of the DPIS. Based on this, it is conceivable to hypothesize that the PFC, the internal descending pain modulation system, and the system controlling the neuroplasticity state are involved in the dysfunction of pain processing pathways and cognitive impairment. We, therefore, postulate that the cognitive performance involving PFC-related neural networks is comparable to the effectiveness of the DPIS and that the tests of working memory, executive function, divided attention, and cognitive flexibility (TMTB-A), as well as the Digits subtest of the Wechsler Adult Intelligence Scale (WAIS-III), are indicators of the severity of cognitive impairment. Therefore, this cross-sectional study sought to respond to two inquiries: (i) To determine whether the assessments of working memory, verbal and semantic fluency, sustained attention, and divided attention can be used to distinguish between cognitive impairment in FM and HC. (ii) To explore in a multivariate hierarchical model the relationship of the impairment in these cognitive tests according to the spectrum of responders and non-responders to the CPM-test, considering the patterns of the severity of symptoms across FM and serum markers of neuroplasticity, nominally the BDNF.

## Materials and methods

### Procedure, study design, and setting

**FIGURE 1:**
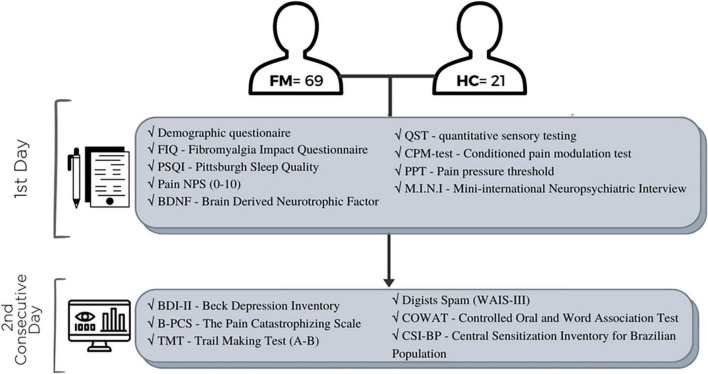
The sequence of assessments.

We performed an exploratory cross-sectional study following the Strengthening the Reporting of Observational Studies in Epidemiology (STROBE) statement. This study has been registered and approved by the Certificate of Presentation of Ethical Appreciation (36995020.3.0000.5327 CAAE registry) and Hospital de Clínicas de Porto Alegre (HCPA) Research Ethical Committee registration number 2017-0330. We performed the research following the Helsinki Declaration. We obtained written informed consent from all participants before taking part in the study. The study enrollment period ranged from May 2018 to December 2021. De-identified data relating to intervention and primary outcomes will be made available on request to WC (wcaumo@hcpa.edu.br) with no time restriction. The sequence of assessments is presented in Figure 1.

### Recruitment, inclusion, and exclusion criteria

#### Fibromyalgia patients

A convenience sample of literate females between the ages of 30 and 65 who had been diagnosed with FM in accordance with the American College of Rheumatology’s 2016 guidelines (Wolfe et al., 2016) was included. They had to report pain scores on the Numerical Pain Scale (NPS) of six or more on most days over the previous 3 months to be considered. They were recruited at the Hospital de Clínicas de Porto Alegre’s (HCPA) Basic Health Unit from the outpatient chronic pain wards, Brazil, and social media. For the initial screening, telephone calls were made to every patient. They were invited for a medical evaluation, medical history taking, a thorough description of their symptoms, and confirmation of the diagnosis if they met the inclusion criteria. Physicians with more than 10 years of expertise in pain management applied the ACR score evaluation. Pregnancy, past alcohol or drug misuse, history of decompensated systemic disorders or any chronic inflammatory condition (such as lupus, rheumatoid arthritis, or Reiter’s syndrome), as well as a personal history of cancer now being treated or in the past 6 months were all exclusion criteria.

We screened 142 eligible participants, 62 were excluded for diverse reasons such as restrictions by pandemic conditions (i.e., infection, fear of contact, difficulty with public transportation, etc.). They did not meet the diagnostic criteria for FM or presented pain levels lower than 6 (NPS 0–10) on most days in the last 3 months. To have another uncompensated clinical disease (rheumatoid arthritis, lupus, hypothyroidism, etc.). We selected 80 participants, remaining a total of 69 subjects. Eleven patients were excluded from the analysis by loss of data, the most part by incomplete cognitive tests because of isolation by the pandemic of SARCS-Cov2.

#### Healthy controls

The group of healthy subjects was chosen from local volunteers. We included literate, healthy women between the ages of 30 and 65. They had a thorough phone screening to make sure they had no serious health problems, were free of any acute or chronic illnesses and were not currently on any medications.

We also screened 22 healthy subjects, and one was excluded for showing a score higher than 13 on the Beck Depression Inventory (BDI-II). However, the final sample comprises 21 individuals.

Demographic characteristics, depressive symptoms, and cognitive performance are presented in Table 1. The analysis showed that FM patients (*n* = 69) are older and have a lower formal education level compared to controls (*n* = 21).

**TABLE 1:** Demographic characteristics and cognitive performance of FM and HC (total *n* = 90).

	Healthy controls (*n* = 21)		Fibromyalgia (*n* = 69)		*P*
	Mean (SD)	Median (IQ_25–75_)	Mean (SD)	Median (IQR_25–75_)	
Age (year) €	45.67 (10.79)	–	49.04 (9.39)	–	0.16
Years of formal education €	16.04 (4.65)	–	12.78 (4.65)	–	0.01
Span digits forward ¥	8 (1.65)	8 (6, 11)	7.33 (2.32)	7 (3, 140)	0.15
Span digits for backward ¥	5.70 (2.02)	6 (2, 9)	5.05 (1.66)	6 (2, 9)	0.16
COWAT-orthographic ¥	33.26 (8.82)	34 (17, 57)	40.95 (9.66)	43 (20, 55)	0.00
COWAT-semantic ¥	16.28 (4.22)	16 (9, 28)	18.30 (3.88)	18 (11, 26)	0.02
Trail Making Test (TMT-A) ¥	29.86 (11.56)	27.60 (14.40, 65.40)	38.99 (15.91)	35.38 (17.67, 122)	0.00
Trail Making Test (TMT-B) ¥	63.05 (23.29)	56.82 (30.20, 125.90)	82.74 (40.340)	72.51 (17.23, 235)	0.02
Trail Making Test (TMTA-B) ¥	80.96 (36.19)	72.38 (17.23;,186.81)	64.32 (23.12)	57.46 (30.20, 125.90)	0.17

€ = Comparisons using t-test for independent sample.

¥ Comparison by Kruskal–Wallis test. 25th to 75th interquartile range [IQR].

### Instruments and assessment of outcomes

#### Dependent and independent variables of primary interest

The dependent variables were sustained and divided attention (TMTB-A), verbal and semantic fluency [Controlled Oral Word Association Test (COWAT)-FAS], and working memory (Digits subtest from WAIS-III). Divided attention (TMTB-A) was the primary outcome, and the WAIS-III and COWAT-FAS were the secondary outcomes used to compare cognitive performance between FM and HC. The main factor of interest was the efficiency of DPMS as determined by the range of respondents and non-responders to the CPM-test.

#### Evaluation of characteristics that are reliant on cognitive performance

To assess distinct aspects of cognitive performance and executive processes, we chose a battery of neuropsychological tests. All tools have been verified for use by Brazilians. Instruments and their properties to identify different dominions of cognitive performance are described below.

(a) The Digits subtest of the WAIS-III consists of eight series of digits that are read aloud to the subject and asked to repeat in the same order (forward) and seven sequences that are asked to repeat in the opposite direction (reverse) (Nascimento, 2004; Wechsler, 2004). The Digits test evaluates working and short-term memory.
(b) The Controlled Oral Word Association Exam (COWAT), known as the verbal fluency test, measures both linguistic and executive processes, such as cognitive flexibility, strategy use, interference suppression, and response inhibition (Perret, 1974; Troyer et al., 1997; Abwender et al., 2001; Hedden and Yoon, 2006; Schinka et al., 2010). Participants in the Orthographic subtest (COWAT-Ort) are asked to list as many words as they can that start with the letters F, A, and S. There is a time limit of 60 s for each letter. Once the root word has been revealed, people cannot use proper names, numbers, or words with multiple tenses or endings (Lezak et al., 2004). The Semantic task (COWAT-Sem) test demands the participant to list the most animals in a certain category as they can. There is a 60-s time limit for this (Lezak et al., 2004).
(c) The Trail Making Test (TMTA-B): This quick, two-part test measures processing speed, split attention, and cognitive flexibility. The subject is directed to trace a line connecting the circled numbers in Part A, which has sequential numbers 1 through 25, to measure sustained attention (Reitan and Wolfson, 1993; Campanholo et al., 2014). The participant is instructed to trace a line connecting circled numbers and circled letters in consecutive order while alternating between numbers and letters (1 to A, A to 2, 2 to B, B to 3, 3 to C, and so on) in Part B, which contrasts with Part A’s alternately mixed numbers and letters (1 to A, A to 2, 2 to B), and it evaluates attention set-shifting (Campanholo et al., 2014). TMTA-B evaluates working memory, executive motor attention, and split attention (Corrigan and Hinkeldey, 1987; Gaudino et al., 1995; Lezak et al., 2004). The amount of time needed to finish the job and the number of mistakes affect how well you score.

#### Evaluation of the independent variables

##### Psychological-physical tests

(d) Quantitative sensory testing (QST) was used to evaluate the heat pain threshold. The thermode was first affixed to the skin of the mid-ventral forearm’s region. The system comprises a Peltier-based thermode (30 × 30 mm) digital device connected to a desktop computer that gauges a person’s tolerance for heat pain using the method of limitations (Schestatsky et al., 2011). The initial temperature was set at 30°C, and it rose by 1°C/s until it reached a high of 52°C. Participants were told to press the button as soon as the stimulation started to be painful. The position of the thermode was gently changed between assessments to prevent nociceptors from becoming sensitized and to prevent the summation effect. With a 40-s interstimulus interval, we conducted three assessments (Schestatsky et al., 2011). For the analysis, we determined the three assessments’ average temperature.
(e) The CPM-test was assessed using the following steps: First, we used the thermode described above in the thermo-test in the non-dominant forearm’s ventral forearm to determine the average of three temperatures measured by the QST for patients’ report scores of 6/10 (NPS, 0–10). Second, after 3 mi, they were instructed to submerge their dominant hand up to the wrist in water that was 0–1°C for 1 min. After 30 s, the QST was introduced to measure the pain score on the NPS (0–10) in the thermo-test area (QST + CPM-test). Third, the difference between the NPS 0–10 at the start of the test (T0) and the pain score on the NPS (0–10) during the cold-water immersion (QST + CPM-test) at the temperature set at 6/10 during T1 was used to determine the CPM-test score. Responders would have values lower than zero whereas non-responders would have a difference in the count on NPS (T1 T0) equal to zero or higher (Botelho et al., 2016; Soldatelli et al., 2021).
(f) Pain pressure threshold (PPT): To conduct the test, we employed an electronic algometer from J-Tech Medical Industries in Midvale, Utah, United States. Patients were advised to distinguish between pressure and pain before the assessment began. Patients were told to verbally communicate their pain when it started. At 3- to 5-min intervals, we took three measures in succession (da Graca-Tarragó et al., 2019).

##### Psychological symptoms, psychoactive drugs, and psychiatric diagnoses

(g) Using the Mini-International Neuropsychiatric Interview (MINI), the psychiatric diagnoses were established (Amorim, 2000). The Diagnostic and Statistical Manual of Mental Disorders (DSM)-III-R/IV and International Classification of Diseases (ICD) 10 criteria are compatible with the MINI, a quick (15–30 min) standardized diagnostic interview that is used in clinical practice and research in primary care and psychiatry. It was certified to identify psychiatric problems in the Brazilian population (Amorim, 2000).
(h) Beck Depression Inventory: This tool was used to evaluate the severity of depressive symptoms (Wang and Gorenstein, 2013).
(i) Brazilian Portuguese Pain Catastrophizing Scale (Br-PCS) was used to assess pain catastrophizing, which is a maladaptive response to pain and is one of the factors that contribute to the chronicity of some pain syndromes (Sehn et al., 2012).

##### Clinical and sociodemographic traits, pain measurements, central sensitization, sleep quality, and overall quality of life

(j) Using a standardized questionnaire, demographic information and medical comorbidities were evaluated. They were asked for their age, gender, number of years of schooling, socioeconomic status, self-reported diagnoses and health difficulties, medication use, medical procedures, and pain-related problems.
(k) A NPS (NPS 0–10) was used to measure the intensity of the pain, zero, no pain, and 10 maximum pain. Patients answered the following question: How severe was your worst pain over the past week?
(l) Central Sensitization Inventory for Brazilian Population (CSI-BP) was utilized to evaluate the central sensitization symptoms. Higher ratings reflect more severe symptoms. Part B of the CSI-BP also evaluates the existence of neurological conditions linked to central sensitization and mental diagnoses (Caumo et al., 2017).
(m) The fibromyalgia impact questionnaire (FIQ) was used to evaluate the quality of life in FM patients. We used the validated version for usage in Brazil (Paiva et al., 2013). There are 10 domains, which comprehend items that evaluate the capacity for doing everyday activities as well as their level of weariness, stiffness in the morning, mood, anxiety, and sadness. The scoring ranges from 0 to 100.
(n) The Pittsburgh Sleep Quality Index (PSQI) was used to rate the sleep quality of the previous month. The PSQI score ranges from 0 to 21. The score with the highest rating represents more severe sleep disturbance.

#### Brain-derived neurotrophic factor evaluation

(o) Dosage of BDNF serum levels: Blood samples were centrifuged and divided into 0.5 ml aliquots for additional examination. According to the manufacturer’s instructions, sandwich ELISA was used to measure the serum levels of BDNF using monoclonal antibodies that are specific for this neurotrophic factor (R&D Systems, Minneapolis, MN, United States). To evaluate the inter-assay variation, two plates per kit were utilized over two distinct days of the same week. The manufacturer’s instructions are followed by protocols. To ascertain serum BDNF, the Enzyme-linked Immunosorbent was employed. The kit’s BDNF lower detection limit is 7.8 pg/ml. Use the ChemiKine BDNF Sandwich ELISA kit, CYT306 (Chemicon/Millipore, Billerica, MA, United States) for the assay (ELISA). GloMax^®^-Multi Microplate Reader from Promega or the Bio-Plex^®^-200 device from Bio-Rad was used to assess optical density for multiplexing assay readings. Using the Bradford method, which uses bovine serum albumin as a standard, we measured the total protein using the standard. The information was presented as pg/mg of protein.

### Actions to address potential bias sources

Two trained psychologists applied the cognitive tests. Each examiner was given a specific training program that included the following steps: (i) Reading and studying each test’s manual; (ii) watching an experienced examiner administer the test; (iii) practicing administering the test on volunteers in role-playing sessions; (iv) if necessary, discussing issues and questions with regional experts. (v) The examiners gave patients accurate instructions for the exam, and all assessments were conducted without interruptions in a quiet, private setting.

### The study sample size

We determined the sample size to equal 39 patients to compare the cognitive performance of FM with HC. The estimation compared the cognitive performance in the Making Test (TMTB-A) based on a prior study. For a Cohen’s d of 0.5, an alpha of 0.05, and a power of 0.80. In this estimation, the averages and standard deviations (SD) for FM and HC were 44.1 (SD, 35.8) and 22.2 (6.5), respectively (Vucurovic et al., 2019). To compare the cognitive performance of respondents and non-responders to the CPM-test, a sample size of 62 subjects was estimated. This estimation was based on a MANCOVA with three levels, five dependent variables, an effect size (f2) of 0.25, an alpha of 0.05, and a power of 0.80 (Soper, 2020). We increased the sample size by 15%, bringing the total to 69 cases. This was done to guarantee the study’s power and prevent the attrition rate from unforeseen events.

### Statistical analysis

Descriptive statistics were presented as mean (standard deviation) or frequency. Age and formal education years were compared between groups using independent sample *t*-tests. The Shapiro–Wilk test evaluates a variable’s normality. The cognitive tests did not meet the criteria for parametric analysis. Thus, we used the generalized linear models (GLMs) to compare the results of cognitive tests (digit span forward and backward, COWAT-semantic and orthographic, TMTB-A) adjusted for years of formal education between groups of FM and HC. We adjusted these cognitive tests by age and education level by using multiple regression models by the stepwise forward method for the exploratory analysis involving the FM subjects, considering this and the consistent evidence that age and years of formal education may influence the performance in cognitive tests (Lenehan et al., 2015).

The adjusted average of digits spans forward, and backward, COWAT-semantic and orthographic, and TMTB-A were included as dependent variables in the hierarchical multilevel multivariate analysis of covariance (MANCOVA). This model was based on an analytic framework defined *a priori*, which integrates the severity of symptoms related to FM according to the spectrum of the responders and non-responders to the CPM-test (Soldatelli et al., 2021). A *p*-value of less than 0.05 on the bivariate analysis presented in Table 4 was required for a factor to be included in the hierarchical multilevel regression model. Another criterion was the biological plausibility that such factors might influence the relationship between DPMS and cognitive performance. The following variables were included in the models based on the biological plausibility despite the *p*-value related to the efficiency of DPMS or cognitive impairment: depressive symptoms, sleep quality, scores on the quality of life due to FM in the FIQ, and the PPT (Silva et al., 2016; Caumo et al., 2017; Soldatelli et al., 2021). Variables were retained in each model level if they had a *p*-value less than 0.05. The first hierarchical level included pain scores, diagnosis of depressive disorders, central sensitization scores, sleep quality, and antidepressant dual and tricyclic. These variables could directly or indirectly determine the effect of all the variables analyzed in the additional analysis hierarchical levels. The second level included pain catastrophizing and depressive symptoms. Finally, the third level had the impact of FM symptoms on quality of life, PPT, and serum BDNF (log). The variables included in the third level are close to the cognitive performance and could have been affected by all the variables studied in the previous hierarchical levels. Following this rationale, we examined the interaction of BDNF with the DPMS function according to responders and non-responders to the CPM-test. The regression coefficients (β) values in Table 5 are derived from the full model with all variables since we do not know the explanatory power of many of these factors across the hierarchy. We used Bonferroni’s Multiple Comparison Test to identify the source of significant differences and adjust for multiple comparisons. For all analyses, we considered a two-sided *p*-value less than 0.05. Data were analyzed using SPSS software version 22.0 (SPSS, Chicago, IL, United States).

## Results

### Evaluation of cognitive function in fibromyalgia and healthy control

Table 2 showed GLM to evaluate the performance in cognitive tests according to groups of FM and HC. The GLM models revealed a statistically significant difference between FM and HC in the Span digits forward (short-term memory), COWAT-orthographic (executive functions related to verbal fluency), and marginally non-significant in the TMTB-A (sustained and alternate attention, and WM). The interaction analysis showed that the lower performing cognitive performance persisted in the FM group despite the years of formal education.

**TABLE 2:** Primary outcomes–generalized linear model analyses to compare cognitive performance in FM and HC (*n* = 90).

Cognitive tests	Beta	SEM	CI 95%	Wald χ ^2^	df	*P*
***Span digits forward***						
(Intercept)	7.534	1.4389	4.71 to 10.35	27.418	1	0.000
Fibromyalgia (*N* = 69)	−3.572	1.5441	−6.59 to −0.54	5.353	1	0.021
Healthy controls (*n* = 21)	0^reference^					
Formal education (years)	0.029	0.0873	−0.14 to 0.20	0.113	1	0.737
*Interaction group × Years of formal education*						
Fibromyalgia	0.253	0.0977	0.06 to 0.44	6.696	1	0.010
Healthy subjects	0^reference^					
***Span digits backward***						
(Intercept)	5.929	1.3158	3.35 to 8.50	20.302	1	0.000
Fibromyalgia (*n* = 69)	−2.465	1.4124	−5.23 to 0.30	3.046	1	0.081
Healthy controls (*n* = 21)	0^reference^					
Formal education (years)	−0.014	0.0798	−0.17 to 0.14	0.033	1	0.856
*Interaction group × Years of formal education*						
Fibromyalgia	0.147	0.0894	−0.03 to 0.32	2.724	1	0.099
Healthy subjects	0^reference^					
***COWAT-orthographic***						
(Intercept)	35.663	6.6631	22.60 to 48.72	28.648	1	0.000
Fibromyalgia (*n* = 69)	−16.451	7.1441	−30.45 to −2.44	5.303	1	0.021
Healthy controls (*n* = 21)	0^reference^					
Formal education (years)	0.279	0.4037	−0.51 to 1.07	0.477	1	0.490
*Interaction group × Years of formal education*						
Fibromyalgia	0.927	0.4511	0.04 to 1.81	4.223	1	0.040
Healthy subjects	0^reference^					
***COWAT-semantic***						
(Intercept)	15.834	3.1312	9.69 to 21.97	25.572	1	0.000
Fibromyalgia (*n* = 69)	−5.412	3.3572	−11.99 to 1.67	2.599	1	0.107
Healthy controls (*n* = 21)	0^reference^					
Formal education (years)	0.163	0.1897	−0.20 to 0.54	0.740	1	0.390
*Interaction group × Years of formal education*						
Fibromyalgia	0.333	0.2120	−0.08 to 0.75	2.464	1	0.116
Healthy subjects	0^reference^					
***Trail Making Test (TMT-A)***						
(Intercept)	51.714	11.143	29.87 to 73.55	21.535	1	0.000
Fibromyalgia (*n* = 69)	4.481	11.970	−18.98 to 27.94	0.140	1	0.708
Healthy controls (*n* = 21)	0^reference^					
Formal education (years)	−1.374	0.6751	−2.69 to −0.5	4.141	1	0.042
*Interaction group × Years of formal education*						
Fibromyalgia	−0.083	0.7587	−1.57 to 1.40	0.012	1	0.913
Healthy subjects	0^reference^					
***Trail Making Test (TMT-B)***						
(Intercept)	50.150	19.622	11.69 to 88.60	6.532	1	0.011
Fibromyalgia (*n* = 69)	40.324	21.087	−1.00 to 81.66	3.657	1	0.056
Healthy controls (*n* = 21)	0^reference^					
Formal education (years)	−1.067	1.1887	−3.39 to 1.26	0.805	1	0.370
*Interaction group × Years of formal education*						
Fibromyalgia	−2.986	1.7043	−6.32 to 0.36	3.069	1	0.080
Healthy subjects	0^reference^					
***Trail Making Test (TMTB-A)***						
(Intercept)	50.150	19.622	11.69 to 88.60	6.532	1	0.011
Fibromyalgia (*n* = 69)	40.324	21.087	−1.00 to 81.66	3.657	1	0.056
Healthy controls (*n* = 21)	0^reference^					
Formal education (years)	−1.067	1.1887	−3.39 to 1.26	0.805	1	0.370
*Interaction group × Years of formal education*						
Fibromyalgia	−2.904	1.3384	−5.52 to −0.28	4.708	1	0.030
Healthy subjects	0^reference^					

### Exploratory analysis of cognitive performance in fibromyalgia subjects

#### Adjustment of cognitive tests performance by years of formal education and age

We adjusted each cognitive test for years of education level and age using linear regression analyses following the stepwise method. The variables age and education level were retained in the regression models only when they correlated with cognitive tests with a statistically significant difference (*P* < 0.05). We found that education level was positively correlated with performance in all cognitive tests. In contrast, the attention and cognitive flexibility tests were negatively correlated with age. However, one needs to realize that the TMTA-B scores assess the time required to complete the task and the number of errors. In this way, higher values indicate the worst performance in the test. So, this result is aligned with the results of other cognitive tests. Table 3 displays the adjusted mean of cognitive tests by age and years of education. We observed that higher prevalence of depressive illnesses in non-responders compared to responders. Additionally, they displayed higher pain scores and a greater degree of pain catastrophizing.

**TABLE 3:** Linear regression analyses to adjust the cognitive performance for years of formal education and age in FM patients.

	B^€^	Std. Error	Beta^¥^	*t*	*P*	CI 95%	Crude mean (SD)	Adjusted mean (SD)
***Dependent Variable: Span digits forward***							7.48 (2.10)	7.30 (1.14)
Formal education (years)	0.341	0.055	0.595	6.152	0.000	(0.23 to 0.45)		
***Dependent Variable: Span digits backward***							5.19 (1.72)	5.04 (0.49)
Formal education (years)	0.108	0.035	0.296	3.130	0.002	(0.04 to 0.18)		
***Dependent Variable: COWAT-orthographic***							34.98 (9.79)	33.56 (4.76)
Formal education (years)	1.082	0.175	0.514	6.176	0.000	(0.74 to 1.43)		
***Dependent Variable: COWAT-semantic***							16.78 (4.39)	16.34 (2.11)
Formal education (years)	0.439	0.081	0.466	5.419	0.000	(0.28 to 0.60)		
***Dependent Variable: Trail Making Test (TMT-A)***							37.82 (5.37)	39.90 (6.43)
Constant	31.40	11.31		2.774	0.007	8.87 to 53.93		
Formal education (years)	−1.13	0.38	−0.31	−2.92	0.004	−1.90 to −0.36		
Age	0.431	0.17	0.256	2.41	0.018	0.076 to 0.79		
***Dependent Variable: Trail Making Test (TMT-B)***							78.77 (38.26)	82.49 (23.83)
Constant	70.65	24.89		2.838	0.006	21.08 to 120.22		
Formal education (years)	−4.41	0.85	−0.47	−5.14	0.000	−6.12 to −2.71		
Age	1.307	0.39	0.307	3.33	0.001	0.52 to 2.09		
***Dependent Variable: Trail Making Test (TMTB-A)***							42.44 (29.050)	43.49 (17.43)
Constant	39.314	19.75		1.99	0.050	−0.016 to 78.64		
Formal education (years)	−3.29	0.682	−0.46	−4.82	0.000	−4.64 to −1.93		
Age	0.876	0.311	0.270	2.82	0.006	0.25 to 1.49		

Data presented the mean (SD) non-adjusted and adjusted mean by formal education and age (n = 69). B€, Unstandardized coefficients; Beta^¥^, Standardized coefficients.

#### Features of fibromyalgia subjects according to responders and non-responders to the conditioned pain modulation-test

Table 4 is presented data FM according to responders and non-responders to the CPM-test. This analysis included 69 patients. However, we found missing data related to the CPM-test in one subject, so the analysis was run with a sample of 68. We observed that non-responders are older and have lower years of formal education. Non-responders compared to responders showed a higher prevalence of depressive disorders. Also, they showed higher scores on pain and a higher level of pain catastrophizing.

**TABLE 4:** Demographic and clinical characteristics of the study sample of FM patients.

Characteristics	Responders (*n* = 42)	Non-responders (*n* = 26)	*P*
Age (years)	47.38 (8.82)	52.73 (8.72)	0.01
Education (years)	13.17 (4.85)	10.67 (4.21)	0.00
American College of Rheumatology (ACR) diagnosis criteria score	22.70 (3.34)	23.24 (4.45)	0.54
Smoking (Yes)	8	14	0.48
Alcohol (Yes)	11	25	0.39
Clinical comorbidity (Yes)	20	46	0.24
Ischemic cardiac (Yes)	1	1	
Hypertension (Yes)	6	24	
Diabetes (Yes)	3	6	
Hypothyroidism (Yes)	7	9	
Asthma (Yes)	2	2	
Other (Yes)	1	4	
**Psychiatric disorder according to the MINI (Yes/No)^†^**			
Maniac-depressive disorder (Yes)	25 (56.8%)	23 (88.5%)	0.00
Generalized anxiety disorder (Yes)	21 (47.7%)	12 (42.2%)	0.59
*Pain, sleep quality and psychological measures*			
Visual Analogue Scale^£^	8.17 (1.18)	8.85 (1.31)	0.01
Beck Depression Inventory II (BDI-II)^£^	25.29 (11.02)	27.38 (10.84)	0.31
Brazilian Portuguese Pain Catastrophizing Scale^£^	34.82 (10.65)	39.19 (8.84)	0.03
Pittsburg Seep Quality Index (PSQI)^£^	12.75 (3.59)	13.42 (93.39)	0.41
Heat Pain Threshold to produce 6/10 on NPS (°C)^£^	37.74 (2.63)	37.32 (3.04)	0.50
Fibromyalgia Impact Questionnaire (FIQ)^£^	68.15 (17.68)	71.11 (16.05)	0.40
Central Sensitization Inventory^£^	63.48 (14.88)	66.59 (14.24)	0.32
Pain pressure threshold (kg/cm^2^/second)^∑^	1.69 (1.49)	1.66 (0.80)	0.24
Change on Numerical Pain Scale during CPM-test^∑^	−2.24 (1.32)	1 (1.19)	0.00
Opioid medication user (Yes)^‡^	12	16	0.17
Acetaminophen (Yes)	12	17	
Dipyrone (Yes)	8	12	
Dorflex (Yes)	16	23	
Opioid medication user (Yes)^‡^	12	16	0.17
Codeine	6	3	
Methadone	0	2	
Buprenorphine	1	0	
Tramadol	5	13	
Active central nervous system medication^‡^	25	42	0.27
Antidepressant tricyclic or dual (Yes)	23	48	0.39
Antidepressant dual (Yes)	18	37	0.26
Antidepressants selective serotonin reuptake inhibitors (Yes)	21	43	0.43
Pregabalin (Yes)	23	43	0.50
Brain-derived neurotrophic factor (BDNF) (ng/ml)	40.58 (27.16)	47.29 (34.10)	0.36
**Cognitive assessments**			
Span digits forward	7.40 (1.10)	6.85 (1.11)	0.05
Span digits backward	5.07 (0.47)	4.84 (0.046)	0.06
COWAT-orthographic	33.88 (4.60)	31.64 (4.64)	0.04
COWAT-semantic	16.48 (2.04)	15.48 (2.03)	0.05
Trail Making Test (TMTB-A)	41.64 (14.76)	50.11 (12.84)	0.03

Values are given as the mean (SD) or number of subjects (n = 68).

^‡^Non-opioid analgesics, opioid analgesics; active central nervous system medications and psychiatric disorder patients could have none or more than one of them.

^∑^Comparison using Wilcoxon Mann–Whitney.

^£^Comparisons using t-test for independent sample.

^†^represents one or more than one psychiatric disorder.

#### Cognitive performance in fibromyalgia according to the spectrum of responders and non-responders to conditioned pain modulation-test

The multilevel MANCOVA was conducted to determine independent factors associated with cognitive tests according to responders and non-responders to CPM-test. Data are presented in Table 5. The MANCOVA model using Bonferroni’s Multiple Comparison Test revealed a significant relationship between the responders and non-responders to CPM-test and cognitive performance (Hotelling’s Trace = 0.29, *F* = (3.30), *P* < 0.01). The variables included in the first hierarchical level were pain scores in the VAS, diagnosis of depressive disorders, central sensitization scores, sleep quality, and antidepressant dual and tricyclic use. The second level included pain catastrophizing and depressive symptoms. Finally, the third level included the impact of FM symptoms on quality of life, PPT, and serum BDNF (log). The regression coefficients (β) values (Table 5) are derived from the full model with all variables. The variables retained in the multilevel model comprise the use of antidepressant dual and tricyclic, the impact of FM symptoms on quality of life, PPT, and serum BDNF (log).

**TABLE 5:** Multilevel MANCOVA to assess the relationship between cognitive performance tests in responders and no responders according to change in NPS (0–10) during the CPM-test and related factors (*n* = 68).

Dependent variable	Type III sum of squares	df	Mean square		*F*		*P*		Partial eta squared
**Corrected model**									
Span digits forward	30.011^a^	6	5.002		5.675		0.000		0.358
Span digits backward	5.468^b^	6	0.911		5.676		0.000		0.358
COWAT-orthographic	512.134^c^	6	85.356		5.509		0.000		0.351
COWAT-semantic	101.039^d^	6	16.840		5.602		0.000		0.355
Trail Making Test (TMTB-A)	6977.627^e^	6	1162.938		5.710		0.000		0.360
**Regression coefficient**									
	***B***	**Std. Error**		***t***		***P***		**CI 95%**	

**Span digits forward**									
Intercept	10.028	1.040		9.642		0.000		(7.94 to 12.10)	
Responders to CPM-test	−2.007	1.198		−1.675		0.099		(−4.40 to 0.39)	
Non-responders to CPM-test	0^reference^								
Use antidepressant dual or tricyclic (Yes)	0.821	0.233		3.525		0.001		(0.36 to 1.29)	
Fibromyalgia Impact Questionnaire (FIQ) scores	−0.015	0.006		−2.334		0.023		(−0.03 to −0.002)	
Pain pressure threshold (kg/cm^2^/second)	0.204	0.091		2.231		0.029		(0.02 to 0.39)	
Brain derived neurotrophic factor (BDNF log) ng/ml	−0.818	0.272		−3.012		0.004		(−1.36 to −0.28)	
Interaction changes on NPS (0–10) during CPM-test vs. serum BDNF (log) ng/ml									
Responders to CPM-test	0.723	0.334		2.168		0.034		(0.06 to 1.39)	
Non-responders to CPM-test	0^reference^								
**Span digits backward**									
Intercept	6.197	0.444		13.961		0.000		(5.30 to 7.08)	
Responders to CPM-test	−0.896	0.511		−1.753		0.085		(−1.92 to 0.13)	
Non-responders to CPM-test	0^reference^								
Use antidepressant dual or tricyclic (Yes)	0.361	0.099		3.633		0.001		(0.16 to 0.56)	
Fibromyalgia Impact Questionnaire (FIQ) scores	−0.006	0.003		−2.171		0.034		(−0.03 to −0.001)	
Pain pressure threshold (kg/cm^2^/second)	0.085	0.039		2.175		0.034		(0.007 to 0.16)	
Brain derived neurotrophic factor (BDNF log) ng/ml	−0.358	0.116		−3.086		0.003		(−0.59 to −0.13)	
Interaction changes on NPS (0–10) during CPM-test vs. serum BDNF (log) ng/ml									
Responders to CPM-test	0.320	0.142		2.245		0.028		(0.04 to 0.60)	
Non-responders to CPM-test	0^reference^								
**COWAT-orthographic**									
Intercept	44.860	4.360		10.288		0.000		(36.14 to 53.58)	
Responders to CPM-test	−8.837	5.024		−1.759		0.084		(−18.88 to 1.21)	
Non-responders to CPM-test	0^reference^								
Use antidepressant dual or tricyclic (Yes)	3.421	0.977		3.501		0.001		(1.47 to 5.37)	
Fibromyalgia Impact Questionnaire (FIQ) scores	−0.060	0.027		−2.264		0.027		(−0.11 to −0.007)	
Pain pressure threshold (kg/cm^2^/second)	0.838	0.383		2.190		0.032		(0.07 to 1.60)	
Brain derived neurotrophic factor (BDNF log) ng/ml	−3.431	1.139		−3.013		0.004		(−5.70 to −1.15)	
Interaction analysis: Changes on NPS (0–10) during CPM-test vs. serum BDNF (log) ng/ml									
Responders to CPM-test	3.138	1.399		2.243		0.029		(0.34 to 5.94)	
Non-responders to CPM-test	0^reference^								
**COWAT-semantic**									
Intercept	21.348	1.921		11.115		0.000		(17.50 to 25.19)	
Responders to CPM-test	−3.967	2.213		−1.793		0.078		(−8.39 to 0.46)	
Non-responders to CPM-test	0^reference^								
Use antidepressant dual or tricyclic (Yes)	1.511	0.430		3.512		0.001		(0.65 to 2.37)	
Fibromyalgia Impact Questionnaire (FIQ) scores	−0.026	0.012		−2.198		0.032		(−0.05 to −0.02)	
Pain pressure threshold	0.381	0.169		2.259		0.027		(0.04 to 0.72)	
Brain derived neurotrophic factor (BDNF log) ng/ml	−1.545	0.502		−3.079		0.003		(−2.54 to −0.54)	
Interaction analysis: Changes on NPS (0–10) during CPM-test vs. serum BDNF (log) ng/ml									
Responders to CPM-test	1.408	0.616		2.285		0.026		(0.18 to 2.64)	
Non-responders to CPM-test	0^reference^								
**Trail Making Test (TMTB-A)**									
Intercept	2.024	15.808		0.128		0.899		(−29.59 to 33.64)	
Responders to CPM-test	31.928	18.216		1.753		0.085		(−4.49 to 68.35)	
Non-responders to CPM-test	0^reference^								
Use antidepressant dual or tricyclic (Yes)	−12.919	3.542		−3.647		0.001		(−20.00 to −5.84)	
Fibromyalgia Impact Questionnaire (FIQ) scores	0.219	0.096		2.271		0.027		(0.03 to 0.41)	
Pain pressure threshold	−2.930	1.387		−2.113		0.039		(−5.70 to −0.16)	
Brain derived neurotrophic factor (BDNF log) ng/ml	12.497	4.129		3.027		0.004		(4.24 to 20.75)	
Interaction analysis: Changes on NPS (0–10) during CPM-test vs. serum BDNF (log) ng/ml									
Responders to CPM-test	−11.456	5.072		−2.259		0.027		(−21.56 to −1.32)	
Non-responders to CPM-test	0^reference^								

^a^R Squared = 0.358 (Adjusted R Squared = 0.295)^a^.

^b^R Squared = 0.358 (Adjusted R Squared = 0.295)_b_.

^c^R Squared = 0.351 (Adjusted R Squared = 0.288)^c^.

^d^R Squared = 0.355 (Adjusted R Squared = 0.292)^d^.

^e^R Squared = 0.360 (Adjusted R Squared = 0.297)^e^.

The performance in the Span Digits (forward and backward) and COWAT (orthographic and semantic) are positively correlated with using antidepressant dual and tricyclic and higher PPT. In contrast, serum BDNF and the negative impact of FM symptoms on the quality of life are negatively correlated with cognitive performance, while serum BDNF was positively associated with the TMTB-A. However, the interaction analysis presented in Table 5 indicates that the BDNF is a moderating factor in the relationship between the cognitive measurement and the dysfunction of DPMS. The beta coefficient of the interaction analysis between BDNF and the spectrum of responders and non-responders to the CPM-test changed the direction of its relationship with cognitive tests. So, these results indicate that the increase in BDNF is positively correlated to more severe cognitive impairment in non-responders.

**FIGURE 2:**
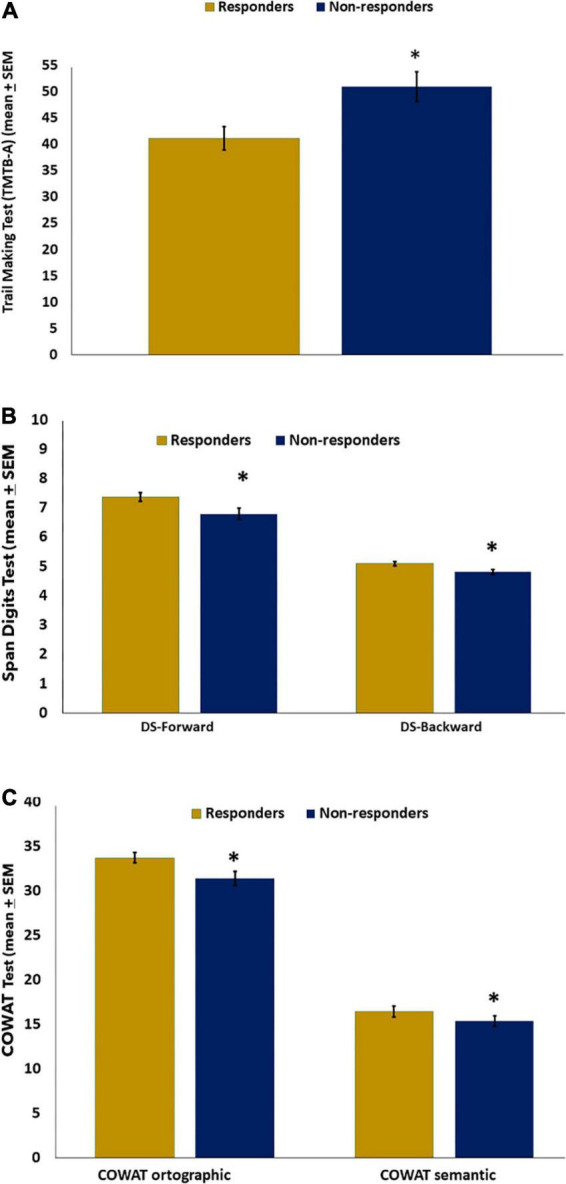
Mean of cognitive performance in FM (*n* = 68) according to responders and non-responders to CPM-test on the Trail Making Test (TMTB-A) **(A)**, **(B)** DS, Digits Spam (Forward–Backward), and **(C)** COWAT-orthographic; COWAT-semantic. The error bars indicate the standard error of the mean (SEM). Asterisks (*) positioned above the bars indicate significant differences (*P* < 0.05). All comparisons were performed by a hierarchical multilevel multivariate analysis of covariance (MANCOVA), followed by the Bonferroni correction for *post hoc* multiple comparisons.

Figure 2A displays results for responders and non-responders to the CPM-test on the TMTB-A (primary outcome). Figures 2B,C showed scores on the secondary outcomes measures according to a spectrum of responders and non-responders to the CPM-test. Working memory tests (DS-forward and DS-backward) were displayed in Figure 2B. Figure 2C presents the scores on executive functions (COWAT-orthographical and COWAT-semantic). The multilevel MANCOVA and Bonferroni’s Multiple Comparison Test were used to compare the means. Table 5 displays the results of the multivariate model. Non-responders need more time to complete the test that measures complex psychomotor-related processing speed, such as divided attention and cognitive flexibility. Non-respondents to the CPM-test compared to responders had lower working memory and executive function scores.

## Discussion

These findings showed that the FM group scored considerably lower on the Span digits forward, COWAT-orthographic, and TMTB-A tests than the HC. The lower performance in cognitive domains, including working memory, attention, and executive function, is related to the deficiency of the inhibitory mechanism of DPMS. We found that BDNF is a moderating factor in the relationship between the severity of cognitive impairment and the dysfunction of DPMS. Antidepressant use and PPT were positively correlated with these cognitive tests. In contrast, the impact of FM symptoms on quality of life was associated with lower cognitive performance.

This study showed that FM has a lower cognitive performance than HC in WM and attention, executive functioning, and information processing speed. Despite the difference between groups in age and education, all analyses were adjusted by these factors, so it is improbable that the confounding effect of these variables explains our results. As previously mentioned, these results converge with studies over the past decade on cognitive impairment in FM, with a special focus on the effects of pain on the key cognitive parameters examined, including attention, learning, memory, sustained concentration, processing speed, psychomotor ability, and executive function (Nadar et al., 2016; Khera and Rangasamy, 2021). Also, they are allied to the results of numerous controlled studies in FM that found impairment in cognitive performance related to attention and memory (Galvez-Sánchez et al., 2018). These findings contradict studies that revealed similar cognitive performance in FM patients and HC, which support the hypothesis that these symptoms are disruptive and disturbing for patients who may report feeling more functionally handicapped by cognitive dysfunction than by pain (Landro et al., 1997; Grace et al., 1999; Leavitt and Katz, 2009; Miro et al., 2011; Walitt et al., 2011; Walteros et al., 2011; Kim et al., 2012; Bar-On Kalfon et al., 2016). The discrepancy among findings may be explained by variation in cognition assessment, involving different neuropsychological tests and distinct methods of applying them. For instance, some studies use computerized experimental tasks, while others use traditional pencil-and-paper neuropsychological examinations (Duenas et al., 2016). The sample size, the severity of the disease, the mental weariness caused by extended cognitive testing, and studies with lower statistical power play a role in this gap. The inability of numerous studies to record psychological symptoms including anxiety, catastrophizing, and self-efficacy may have an impact on cognitive function and chronic pain. Additional factors, such as sleep disruption and psychotropic medications, such as antidepressants, anticonvulsants, opioids, etc., might interact with chronic pain and cognitive function (Menefee et al., 2000).

This study is significant because it is the first to show a connection between cognitive decline and DPMS dysfunction. Given the exploratory nature of our study, we were unable to establish a cause-and-effect relationship between cognitive symptoms related to the PFC neural networks with deficiencies in the DPMS pathways due to the exploratory nature of our study. It is also possible that both processes are effects of distinct phenomena. The current research, however, showed that this integrative pattern evaluates changes that might affect cognitive function and aberrant pain pathway activities. They emphasize how BDNF secretion, the primary indicator of neuroplasticity, changes along with the interaction between the neural network comprising cortical areas and the spine-bulbospinal loop. This modification thus supports the idea that the DPMS and the brain networks involved in cognitive processing have similar neurobiological underpinnings. Given that FM is a condition of nociplastic pain, central sensitization is its primary underlying mechanism (Kosek et al., 2021). Therefore, a characteristic of the clinical picture known as central sensitization syndrome may be the degree of cognitive impairment (Caumo et al., 2017). Altered activity in brain-orchestrated nociceptive facilitatory pathways is one of the mechanisms underlying these various dysfunctions of the central nervous system (Staud et al., 2008; Bosma et al., 2016). It includes the dysfunction of the DPMS and brain areas involved in processing sensory and cognitive information, such as the insula, ACC, and PFC (Nijs et al., 2021). Because of this, these findings provide a neurobiological substrate to link the clinical symptoms that make up the “FibroFog” with the variation in the dysfunction spectrum of DPMS, even though the underlying mechanisms are not fully understood. Responders and responders to the CPM-test presumably differ regarding an imbalance between systems involved in excitability and inhibition in pain pathways. Pre-clinical studies indicate that this imbalance is biologically plausible because peripheral inflammation dynamically upregulates both BDNF in the PAG and its receptor TrkB in the rostral ventromedial medulla (RVM), which plays a central role in initiating and maintaining neuronal hyperexcitability. This may help explain how the BDNF may be crucial for initiating and maintaining the maladaptive neuroplasticity that underlies persistent pain and its relationship to DPMS dysfunction and cognitive decline (Guo et al., 2006).

Brain-derived neurotrophic factor is a moderating factor in the relationship between cognitive measurements and the dysfunction of DPMS. In non-responders, the rise in BDNF is positively correlated with more severe cognitive impairment. Although the design of this study leaves it unclear whether alterations in serum BDNF were caused by the disease or occurred before cognitive dysfunction in FM, these findings help to strengthen the case that BDNF functions as a moderator in this association. Even though they are pertinent to understanding in a functional framework the mechanism that connects functions involving PFC neural networks with the DPMS, we cannot confidently say whether the clinical outcome is related to changes in this neurotrophic factor in a particular neural network. This integrative view gives support to understanding the effects of interventions that can improve pain and enhance cognitive performance [i.e., antidepressant duals and tricycle, transcranial direct current stimulation (tDCS), transcranial magnetic stimulation (TMS)] (Nir et al., 2012; Nahman-Averbuch et al., 2016). However, we are unable to determine if these changes are related to persisting chronic pain conditions, such as mirroring pain-related stress, inactivity, depression, and inadequate sleep quality (Bäckryd et al., 2017) or if these neuroplasticity marker increases are linked to the severity of clinical symptoms, including the dysfunction of DPMS (Botelho et al., 2016; Soldatelli et al., 2021). Even though there is growing literature about BDNF as a marker of the severity of clinical symptoms of FM (Alves et al., 2020), serum levels are an indirect measure of the BDNF of the brain since it contributes to 70–80% of circulating BDNF (Poduslo and Curran, 1996; Pan et al., 1998; Laske and Eschweiler, 2006). So, longitudinal studies are required to conclude if the generation of BDNF is a compensatory mechanism related to maladaptive neuroplasticity due to the severity of central sensitization or if it is a driving force underlying neuroplasticity involved in neural repair, or both.

Additionally, our results show that FM patients who take dual antidepressants performed better on cognitive tasks that measured cognitive flexibility, speed, and divided attention assessed by the TMTB-A test. In this situation, the advantages of antidepressant dual may be linked to improvement in the DPMS, improvement of depressive symptoms, or both. This hypothesis found support in an earlier study that dual antidepressants might improve the efficiency of DPMS (Marks et al., 2009; Bidari et al., 2019). The fact that FM is a complex syndrome with additional confounding factors that cannot be controlled entirely must be considered. Polypharmacy is just one of the many contributing factors that FM patients typically experience throughout their lifetimes of chronic suffering. Despite the drawbacks of an exploratory study, these findings represent the clinical profile of FM patients and add to the neurobiological foundation for the connections between the DPMS and cortical areas involved in cognitive processing.

Pain pressure threshold and cognitive function have a positive relationship aligned with the idea that allodynia and hyperalgesia are reflected in pain responses to low pain thresholds. Since pain is an attention-demanding condition that lowers the brain resources available for cognition, this result may be read as an interference impact of pain on attention and cognitive functioning. According to research (del Paso et al., 2012), there is some overlap between the brain networks that control attention, memory, and executive functions and those that control how pain is processed (de Guevara et al., 2018). Although we lack a clear explanation for the positive link between PPT and cognitive ability, we must remember that the groups we are comparing have varying degrees of DPMS dysfunction. As a result, it is conceivable that fewer cognitive resources were available during experimental pressure stimulation due to increased demands on central nervous system resources (Duschek et al., 2012; Montoro et al., 2015). According to PPT, the severity of cognitive dysfunction related to pain interferes with the standard performance of several activities, including movement, leisure activities, sleep, self-care, housework, job, and psychological functioning. The converse relationship between cognitive performance and the worst quality of life is also in line with this perspective (Silva et al., 2016).

We addressed some points concerning study design that should be considered. First, because the cognitive impairments (such as concentration, multitasking, memory, attention, executive function, etc.) are either the same or different in other chronic pain illnesses, a related drawback involves the unsure representativeness of the present sample. Therefore, this must be used to interpret these results. Second, we believe this sample size is appropriate to confirm the clinical significance of the reported results. However, due to the exploratory nature of the correlation study and the considerable number of computed correlations, which increases the likelihood of alpha mistakes, care must be used when interpreting the findings. Third, we only included females since there are sex differences in how pain is processed, involving physiological and psychological factors, including the excitability in the corticospinal pathway, the capacity to withstand pain, pain expectation, etc. (Wiesenfeld-Hallin, 2005; Gasparin et al., 2020). Fourth, psychiatric illnesses are an uncontrollable potential confounding factor in cognitive function in chronic pain. It’s also important to note that our control sample was, on average, younger and had a higher level of formal education. Because of this, we run all analyses using the adjusted mean for education level to avoid the impact of these confounding factors.

These findings showed that HC performed substantially better on cognitive exams than FM did. They demonstrated a link between clinical complaints about attention and memory and decreased DPMS effectiveness. Additionally, they showed that BDNF is a moderating element in the relationship between the severity of cognitive impairment and DPMS dysfunction.

## Data availability statement

The raw data supporting the conclusions of this article will be made available by the authors, without undue reservation.

## Ethics statement

The studies involving human participants were reviewed and approved by Hospital de Clínicas de Porto Alegre (HCPA) Research Ethical Committee registration number 2017-0330. The patients/participants provided their written informed consent to participate in this study.

## Author contributions

PS, RA, and GB participated in the sequence alignment, participated in the design of the study, drafted the manuscript, and approved the final version to be published. MZ participated in the sequence alignment, participated in the design of the study, and approved the final version to be published. CD and AM participated in the sequence alignment and approved the final version to be published. IT and FF drafted the manuscript and approved the final version to be published. WC conceived the study, performed the statistical analysis, participated in the design of the study, drafted the manuscript, and approved the final version to be published.

## Abbreviations

ACC: anterior cingulate cortex
ACR: American College of Rheumatology
BDI: Beck Depression Inventory
BDNF: Brain-derived neurotrophic factor
Br-PCS: Brazilian Portuguese Pain Catastrophizing Scale
ICD: International Classification of Diseases
COWAT: Controlled Oral Word Association Test
CPM-test: conditioned pain modulation test
CSI-BP: Central Sensitization Inventory for Brazilian Population
DLPFC: dorsolateral prefrontal cortex
DNIC: diffuse noxious inhibitory control
DPIS: descending pain inhibitory system
DPMS: descending pain modulatory system
DSM: Diagnostic and Statistical Manual of Mental Disorders
FIQ: fibromyalgia impact questionnaire
FM: fibromyalgia
GLM: generalized linear models
HC: healthy controls
mPFC: medial prefrontal cortex
NPS: Numerical Pain Scale
PAG: periaqueductal gray
PFC: prefrontal cortex
PPT: pain pressure threshold
PSQI: Pittsburgh Sleep Quality Index
QST: Quantitative sensory testing
STROBE: Strengthening the Reporting of Observational Studies in Epidemiology
TMT: Trail Making Test
WAIS-III: Wechsler Adult Intelligence Scale
WM: working memory.
